# The brave new world of artificial intelligence: dawn of a new era

**DOI:** 10.1016/j.igie.2023.01.008

**Published:** 2023-02-28

**Authors:** Giovanni Di Napoli, Linda S. Lee

**Affiliations:** 1 Gastrointestinal Operating Unit, Medtronic; 2 Division of Gastroenterology, Hepatology and Endoscopy, Brigham and Women’s Hospital, Boston, Massachusetts, USA

## Editor’s Introduction

**Figure 1:**
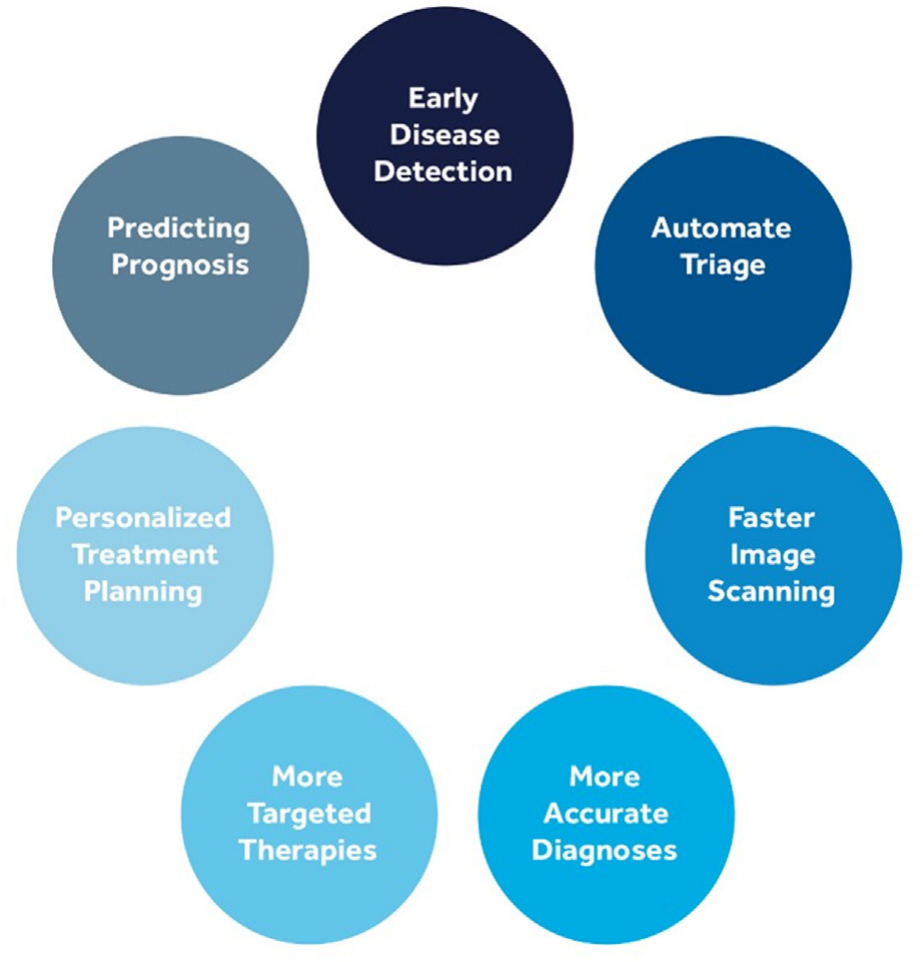
Artificial intelligence in health care.

“It will either be the best thing that’s ever happened to us, or it will be the worst thing. If we’re not careful, it very well may be the last thing.” These were the late Stephen Hawking’s stark words concerning artificial intelligence (AI). The term was coined in the summer of 1956 during a Dartmouth conference of computer scientists including John McCarthy, one of the founding fathers of AI. Thus began the field of AI that, however, experienced several fits and starts throughout the subsequent decades. It was not until the 21st century with the advent of big data, cheap and fast computers, and advanced machine learning that AI really exploded. Currently, it permeates nearly every aspect of our daily lives from Siri, Alexa, Roomba, Dragon, to chatbots and will only grow in its influence over our lives. Adoption of this technology in medicine initially focused on dermatology and ophthalmology with recent spread to nearly every area of medicine, including gastroenterology (Fig. 1).

A significant milestone in 2020 was the Centers for Medicare & Medicaid Services approving reimbursement of 2 AI systems: ContaCT (Viz.ai, San Francisco, Calif, USA), a computer-assisted system that alerts a neurosurgeon when evidence of a blood clot is detected in the brain on CT, and IDx-DR (Digital Diagnostics, Oakdale, Iowa, USA), a system that analyzes photos of retinas to diagnose diabetic retinopathy. Because new technology uptake often remains muted without reimbursement, this was important as typically private insurance companies take their cue from the Centers for Medicare & Medicaid Services paying for their Medicare and Medicaid patients.

Another important factor in adoption of AI technology for clinical use is, of course, obtaining approval from the U.S. Food and Drug Administration (FDA). A 2020 study noted an accelerated pace of FDA approval for machine learning technology particularly starting in 2019.^1^ Of note, most of these have FDA clearances rather than approvals. FDA approval requires demonstration that the benefits of the product outweigh the risks, and the company must submit a premarket approval application with clinical study results. Class III medical devices, which are any implants such as heart valves and breast implants, require FDA approval. Class I and II are low-risk devices used outside the body, such as catheters, Apple’s electrocardiogram app, and motorized wheelchairs. These devices are given clearance by the FDA, which requires the company to submit a premarket notification submission or 510(k) and demonstrate that their product is “substantially equivalent” to another similar device that already has FDA clearance or approval.

Of course, the problem with a new product without any predicate is that it would automatically be classified as a class III device. However, the 1997 FDA Modernization Act provided for a de novo pathway for low- to moderate-risk devices, and a 2012 amendment to the 513(f)2 Federal Food, Drug, and Cosmetic Act allows manufacturers to apply for de novo classification without submitting a 510(k). These products have no equivalent device on the market. GI Genius (Medtronic, Sunnyvale, Calif, USA) was the first endoscopy-related AI product to receive de novo FDA clearance on April 9, 2021 to perform computer-aided detection (CADe) of colon polyps. Because colorectal cancer remains the second leading cause of cancer-related death in the United States and the world,^2^ efforts to improve our screening tools are important especially as an endoscopist’s adenoma detection rate (ADR) is inversely related to risk of interval colon cancer.^3^

Because Medtronic brought the first FDA-cleared AI device for clinical use in endoscopy, I am delighted to have a conversation with Giovanni Di Napoli, President of the Gastrointestinal Operating Unit within the Medical Surgical Portfolio at Medtronic. He has been with Medtronic for over 15 years and was recently awarded the prestigious Crystal Award from the American Society for Gastrointestinal Endoscopy in recognition of his contributions and commitment to new technologies that improve patient care and reach. Gio earned a BS in Business Administration from University La Sapienza in Rome, Italy and an MBA from Luigi Bocconi Business School in Milan, Italy. Before joining Medtronic, Gio held leadership positions in different divisions at Johnson & Johnson and Ethicon. Gio lives in San Jose, California with his wife and 2 daughters.

**Figure 2:**
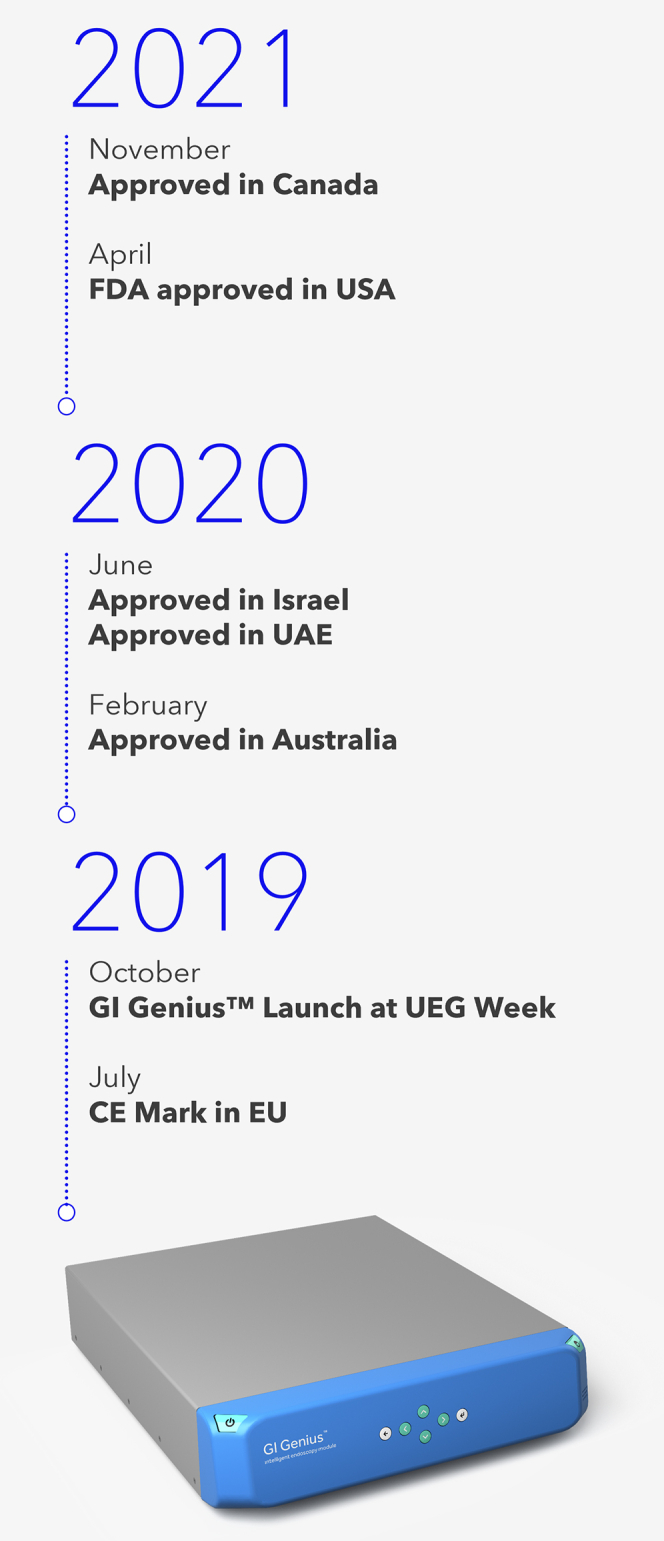
Timeline of key regulatory events in FDA de novo clearance of GI Genius. *FDA*, U.S. Food and Drug Administration; *UAE*, United Arab Emirates; *UEG*, United European Gastroenterology; *CE*, Conformite Europeenne; *EU*, European Union.

**Linda Lee (LL):** Gio, thank you very much for making time with your incredibly busy schedule to discuss AI in GI. Would you discuss why Medtronic decided to pursue developing AI for use during colonoscopy? (Fig. 2).

**Giovanni Di Napoli (GDN):** Colonoscopy is a commonly used diagnostic tool in gastroenterology, but it has challenges, and the detection of difficult polyps is one of many that are important in colorectal screening. The data show that there’s wide variability in physician performance and therefore procedural outcomes. Much of this has to do with the very human limitations we all have—fatigue, distraction, and so on.^4^ The development of AI technology for colonoscopy could help to improve the overall efficiency and effectiveness of the procedure. Medtronic is bringing the first real-time AI medical device in endoscopy to the market, demonstrating its commitment to innovation.

**Figure 3:**
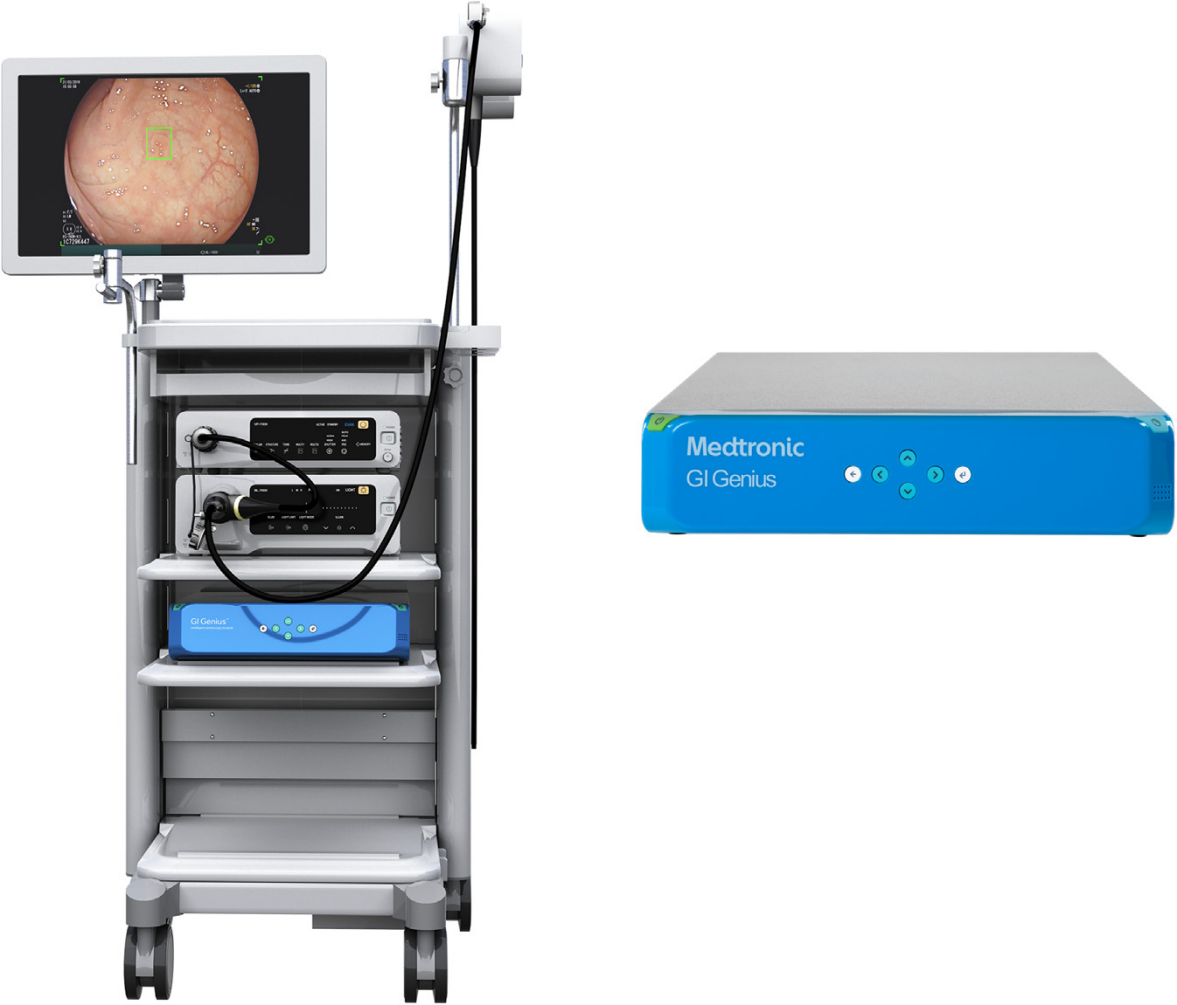
GI Genius system.

**LL:** What were the challenges you faced at Medtronic as you developed GI Genius? (Fig. 3).

**GDN:** GI Genius was developed by Cosmo AI/Linkverse, part of the Cosmo Pharmaceuticals(Dublin, Ireland) group and a strong partner of Medtronic. Several challenges were addressed while developing GI Genius. Some of the key challenges included the following:

• Data availability and quality: A critical requirement for developing an AI-enhanced medical device is obtaining a large, diverse, and high-quality dataset required to develop and train the AI algorithm to analyze medical videos accurately. However, obtaining sufficient and high-quality data can be challenging, mainly if the data must be collected from multiple sources or if there are privacy or regulatory constraints. Cosmo AI leveraged their considerable experience as a pharmaceutical group in handling international, multicenter, clinical studies with thousands of patients. In addition, the quality of procedures was ensured by including some of the top endoscopists in the data collection effort.
• Algorithmic complexity: Medical videos can be complex and varied, and it can be challenging to develop algorithms that can accurately and reliably analyze such data in real time. This complexity requires using advanced machine learning techniques and developing specialized models that can handle the unique characteristics of medical video data.
• Integration with existing systems: An AI-enhanced medical device for endoscopy must integrate seamlessly with current procedures and endoscopes to be effective. This can be a complex and time-consuming process, particularly if the device must interface with various systems.
• Regulatory considerations: Medical devices, including those that use AI, are subject to strict regulatory requirements. Any AI-enhanced medical device must be thoroughly tested and evaluated to ensure it meets all relevant safety and effectiveness standards. Regulations for AI-based medical devices are still evolving because it is an ever-evolving technology.
• Ethical and social considerations: The use of AI in health care raises several ethical and social concerns, including issues related to privacy, equity, and bias. It is essential to carefully consider these issues when developing an AI-enhanced medical device and ensure that the technology is being used ethically and responsibly.
• Clinical validation: To be used in a clinical setting, an AI-enhanced medical device must be validated by well-designed clinical studies to demonstrate its effectiveness and safety. This can be a time-consuming and resource-intensive process.

**LL:** What hurdles were unique to bringing this system to the United States compared with the rest of the world?

**GDN:** Some of the main challenges that Medtronic encountered explicitly for the United States included the following:

• Regulatory requirements: In the United States, medical devices incorporating AI technology are subject to rigorous regulatory oversight by the FDA.
• Reimbursement challenges: In the United States, medical devices are often reimbursed by private insurance companies or government programs, such as Medicare and Medicaid. To be eligible for reimbursement, a device must be approved by the FDA and demonstrate clinical effectiveness and cost-effectiveness. This can be a challenging process, particularly for innovative technologies like AI-enhanced medical devices.
• Competition: The United States is a large and highly competitive medical device market with many well-established players. Medtronic is a leader in AI-enhanced endoscopy and is committed to remaining so. That being said, we are aware that the United States is also home to many leading healthcare institutions and is a major market for medical devices, so it can also be a very attractive market for companies looking to sell AI-enhanced medical devices.

**Figure 4:**
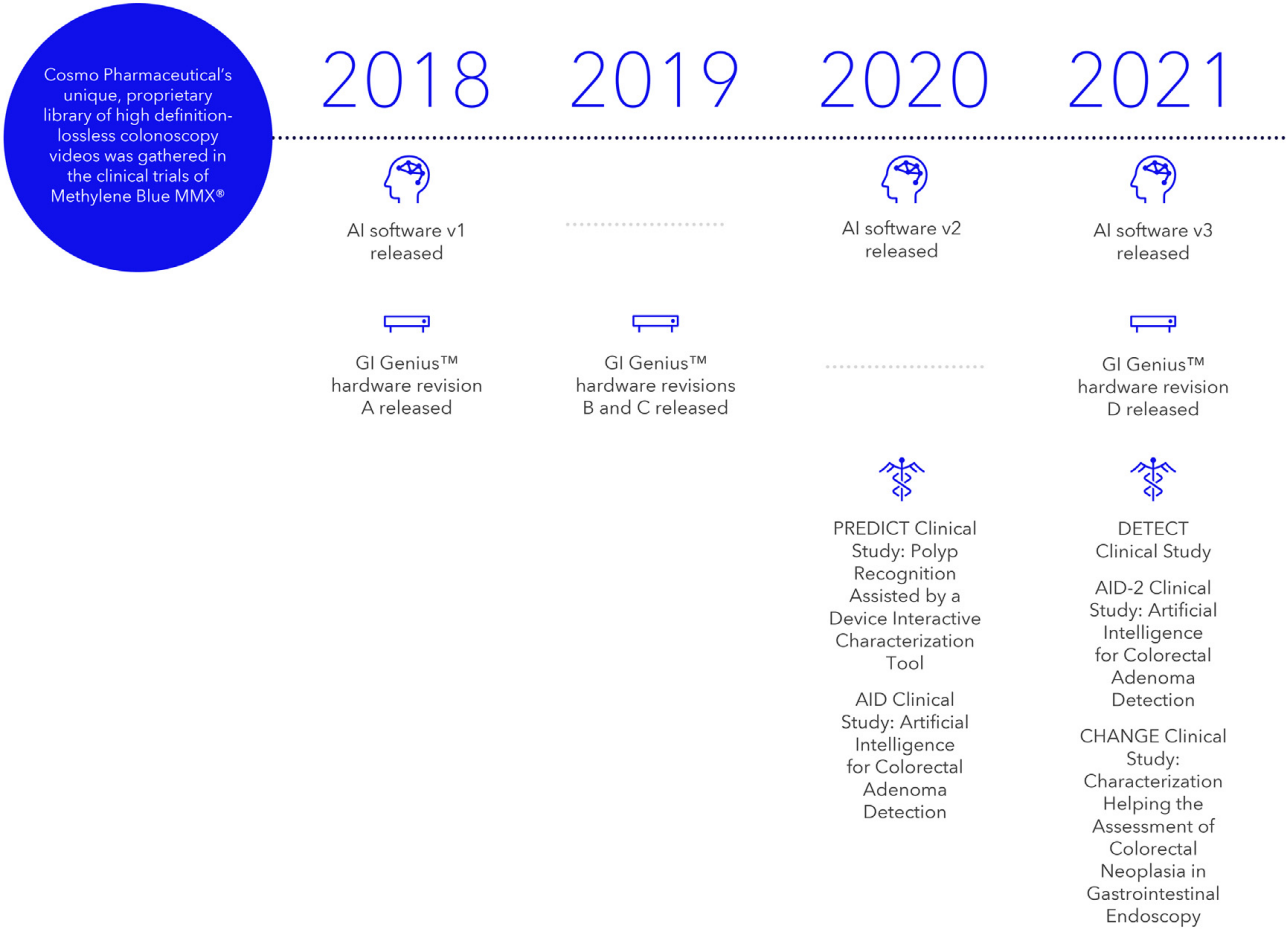
Key steps in the development of GI Genius. *AI*, Artificial intelligence.

**LL:** How was this technology developed? What were the steps involved in taking it from a concept to clinical use? (Fig. 4).

**GDN:** GI Genius was developed entirely by our partner Cosmo AI/Linkverse, part of the Cosmo Pharmaceuticals group. Medtronic's mission to restore health and extend life is a perfect match for Cosmo's mission to improve clinical outcomes in the field of endoscopy. To these aims, when Cosmo discussed its project in the field of AI with Medtronic and later showed the first GI Genius prototypes, which were the result of Cosmo's research and development efforts in the area of colonoscopy, it was natural to establish the basis for a very fruitful relationship, where Medtronic could market the current and future intelligent medical devices developed by Cosmo.

Cosmo started the development of GI Genius in 2013 when they began collecting data in a way no other company had been collecting them, even today. Data are composed of clinical data and video. Clinical data have been collected with very tight control in the whole lifecycle—more than was done even for a phase III study in endoscopy—bringing the drug standards to this field for the first time, and full videos have been recorded in a lossless format, a mode that ensures no details are lost. A recorder with such features did not exist then, and Cosmo had to design and develop it to allow this recording capability.

More specifically, the GI Genius CADe module has been trained using data collected during a clinical study sponsored by Cosmo Pharmaceuticals that included CRC screening and surveillance patients undergoing colonoscopy.^5^ The study involved key opinion leaders in the European Union and United States—the best endoscopists you could find at the time, so we had the privilege of fully recording their performances. Their ability to find lesions in the colon was key to building a massive database of all types of lesions, from those that are easy to find to those that are very difficult. The efficacy of GI Genius in increasing ADR and reducing polyp miss rates was later proven by different randomized controlled trials.^6^^,^^7^

So top-class data from top-class endoscopists. What's next?

Cosmo had to build a hardware platform. Here again, the vision has been crucial. Cosmo understood that it had to bring breakthrough innovation, so it created a platform that could deliver best-in-class performances. To do so, Cosmo arranged to get the most powerful and flexible technology to give its full thrust to the algorithms it would then have implemented. Therefore, they designed and developed a solution to deliver all this in the endoscopy room conveniently. None existed at the time.

LL: So top-class data from top-class endoscopists and a breakthrough platform.

GDN: Cosmo has a deep background in GI and has always been looking to improve the standard of care. Platform engineering is something you design and build comprehensively, and it cannot be done without understanding the exact mechanisms involved in discovering polyps during a colonoscopy.

**Figure 5:**
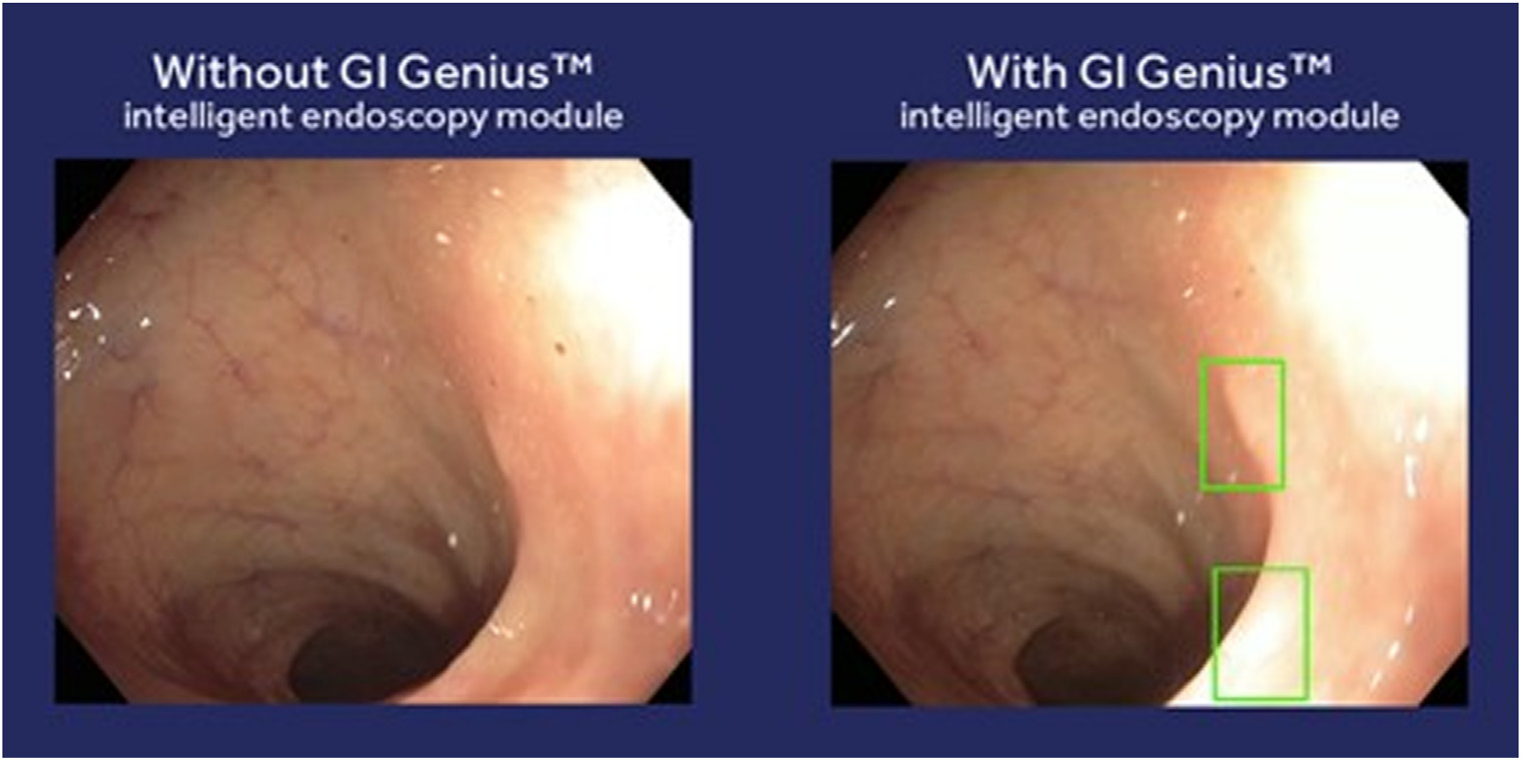
Colonoscopy images with and without GI Genius.

**LL:** What is Cosmo doing to improve the technology, including issues of false positives? (Fig. 5).

**GDN:** Cosmo is constantly improving the technology behind GI Genius, paving the way for introducing novel features and ameliorating the performances of the current features, such as polyp detection or CADe.^8^^,^^9^ Cosmo performed several experiments regarding false positives to optimize the GI Genius CADe working point between sensitivity and specificity. Multiple scientific evidence proves that:

• The absolute rate of frames containing a false positive is less than 1% of the total number of frames.^10^
• A study measuring how many of them were judged of "clinical relevance" (ie, required further inspection by the endoscopist) counted 1 to 2 false positives per procedure, accounting for less than 5 additional seconds of inspection time.^6^^,^^11^
• In 2 randomized controlled trials, there was no statistical difference in withdrawal times between the arm using GI Genius and the arm without AI.^12^^,^^13^

Although the number of false positives is meager, Cosmo is committed to further improving the detection performances of GI Genius CADe, pushing the limit of specificity to even lower false-positive rates and the timely detection of even more difficult-to-spot colon polyps. This is achieved by the vast expansion of datasets for AI training and the evolution of new-generation AI algorithms for polyp detection and implemented via software updates.

**LL:** With all the potential benefits from AI, what do you see as some downsides that we need to be aware of as we adopt this technology for clinical use?

**GDN:** Not all AIs are created equal, and it is of paramount importance to introduce into clinical use only those devices whose effectiveness has been demonstrated by well-designed and top-level clinical studies. I hear from the endoscopic community fears of *over-reliance* on the AI output that could bring a phenomenon of *underskilling*, that is, losing the ability to perform procedures.^14^ History teaches us that these fears appear every time a new technology interacting with human abilities becomes available. However, there’s very little experimental evidence to support these fears. In fact, a fascinating study comparing endoscopists' performances with and without GI Genius appeared recently in *Scientific Reports* and showed significantly different behavior.^13^ In this study, there was no over-reliance. The endoscopists were clearly able to retain their decision when correct and follow the AI advice when appropriate, reaching very high accuracies.

Finally, I would like to mention a topic essential for Medtronic. There is a risk that these innovative technologies will not reach underserved communities. To properly tackle this challenge, the Medtronic Health Equity Assistance Program for colon cancer screening in medically underserved communities across the United States, with support from Amazon Web Services' Health Equity Initiative, has donated 133 GI Genius units to 62 facilities. The American Society for Gastrointestinal Endoscopy selected facilities to receive the free equipment with the hopes of helping more than 350,000 patients. This effort is part of Medtronic's Zero Barriers approach to building equity within our healthcare system, accelerating our innovation, and bring our lifesaving technologies to more patients. The program represents a continuation of Medtronic's commitment to health equity anchored in healthcare technology.

**LL:** How do you think hospitals and physicians will be able to justify the extra cost of AI systems? Do you see reimbursement happening for AI technology used clinically?

**GDN:** I believe the scientific and clinical evidence clearly shows the advantage of using GI Genius. Miss rates are dropping, and ADR increases when GI Genius is used. After the first landmark randomized controlled trials, there is now confirmation of GI Genius' effectiveness coming from real-world practices. I believe that, as with all novel technologies, the practice of reimbursement for AI-enhanced endoscopy is being carefully considered, and rightly so. However, I see no possibility that AI will not be included in future reimbursement procedures.

**Figure 6:**
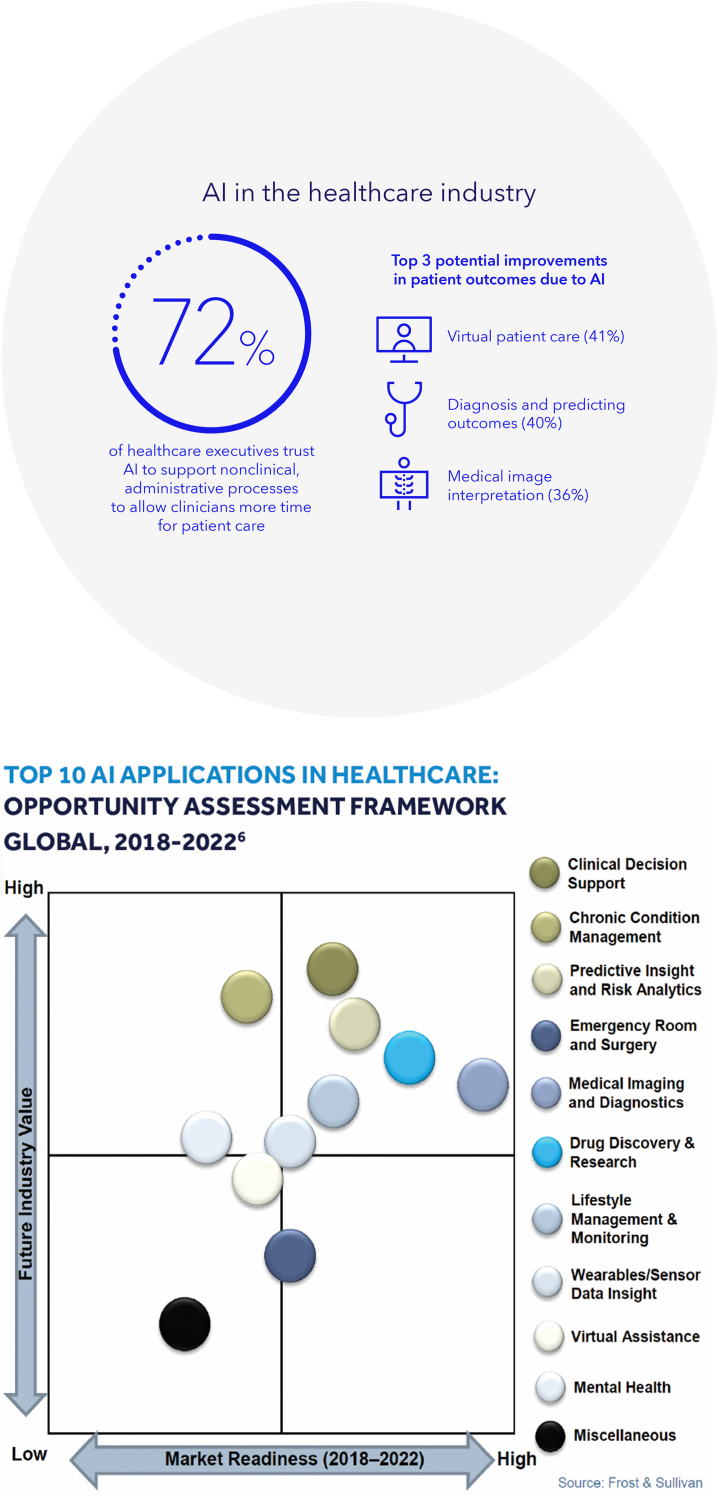
Opportunities for AI in health care. *AI*, Artificial intelligence.^15^^,^^16^

**LL:** What other areas is Medtronic exploring for use of AI including its use with other clinical devices such as Endoflip, EUS, but also in other areas outside of procedures such as doing reports, assisting with colonoscopy preparation, etc? With so many potential options for AI in all aspects of medicine, how is Medtronic prioritizing which areas to focus on next? (Fig. 6).

**GDN:** Medtronic considers several factors when deciding which medical areas to focus on in developing AI technologies. In particular, we prioritize developing AI technologies for medical areas where there is a high unmet need or where the technology can significantly improve patient outcomes.^15^^,^^16^ Naturally, we can leverage a strong expertise in real-time AI for medical video, a vast base of GI Genius installations, strong relationships with clinical partners, and access to large amounts of relevant data. In general, we will always keep our mission and vision front and center: We improve patient outcomes by empowering every gastroenterologist across the globe with AI and disruptive therapeutic technologies.

## Editor’s Closing Remarks

FDA clearance of the first AI-device for CADe of colon polyps from Medtronic has ushered in an exciting new chapter in GI and endoscopy with already 2 more FDA cleared CADe systems in the US (EndoScreener, Wision AI Ltd and Micro-Tech, Mich, USA and SKOUT, Iterative Scopes, Inc, Mass, USA). Issues and questions remain, including cost and utility. Although some modeling studies suggest AI during screening colonoscopy is cost-effective,^17^ companies will have to consider how best to package AI devices and whether incorporating them as standard equipment with scopes and processors makes the most sense. This would be analogous to the trend with automobiles where more and more safety features are now standard rather than options to be purchased at additional cost.

While a metanalysis demonstrated that AI is superior to other ADR-enhancing devices including caps and Endocuff (Olympus America Corp, Center Valley, Pa, USA), more studies are needed comparing AI with other ADR-enhancing strategies. Also, despite initial exciting results, a recent retrospective study at a large academic medical center and randomized trial in the United Kingdom of CADe (GI Genius) noted no difference in ADR between CADe use and standard colonoscopy.^18^^,^^19^

Beyond financial issues, there remain other concerns related to AI use in medicine that must be addressed. Concerns around datasets used to generate the AI algorithms include ensuring generalizability and equity while minimizing bias to avoid further marginalizing minorities. Developing a large, labeled database of images and videos requires experts to review and label the images and videos, which is a challenge especially for less-common diseases. Patients’ privacy must be protected in these datasets as well. Also, models continually learn from new data over time, which leads to updates to the algorithms. Although traditionally only the initial model was approved as a locked algorithm, the FDA has been working on a process to allow a model to iterate and adapt without needing repeated FDA clearance.^20^

An important area of concern is the impact of AI on physicians: Will physicians become too reliant on AI, not learn fundamental skills, and lose their skills? By relying on CADe to identify all polyps, might the endoscopist become complacent in performing meticulous mucosal examination? Or if new trainees learn how to do colonoscopy and identify polyps with AI, will they not know how to characterize or even identify different polyps without AI? Or will this not matter akin to our not needing to learn the times table anymore with calculators on our ubiquitous smartphones calculating tips, etc? An example closer to home is the long-overdue drive to change the country’s reliance on the archaic, costly, 10-year recertification sit-down examination (eg, learning Paris classification of polyps) because in reality we practice by querying online to find the latest papers, guidelines, and research about various clinical issues and consulting with our colleagues (eg, computer-aided diagnosis predicts polyp classification and pathology). An equally important issue is accountability: If the AI algorithm makes a mistake, who is liable?

With many different groups promulgating their own AI algorithm for improving detection of various lesions throughout the GI tract including Barrett’s esophagus, gastric cancer, inflammatory bowel disease, and biliary strictures, there must be careful, thorough development and validation of algorithms through multicenter prospective studies. To ensure AI algorithms meet appropriate criteria for clinical use, the European Society of Gastrointestinal Endoscopy recently published their position statement defining performance outcomes for AI in diagnosing various GI neoplasia as being comparable with expert endoscopists.^21^ Also, the potential impact of AI in periprocedural arenas, such as documentation, assisting with and assessment of colonoscopy preparation, scheduling, and triaging, is vast and would bring significant benefits. Although the future appears bright, many areas of uncertainty remain with AI being in its infancy in endoscopy.

## Disclosure

*The following authors disclosed financial relationships: G. Di Napoli: Employee of Medtronic; board member for Palliare and Aqua Therapeutics. L. S. Lee: Consultant for Boston Scientific, Fractyl, and Fujifilm Medical; research* *support* *from Fujifilm Medical.*
